# Disitamab vedotin *vs*. gemcitabine-cisplatin regimen with immunotherapy: a comparative analysis of efficacy and safety in muscle-invasive bladder cancer

**DOI:** 10.3389/fimmu.2025.1549647

**Published:** 2025-02-27

**Authors:** Chuanao Zhang, Yanhang Yu, Qi Zhou, Jun Ouyang, Zhiyu Zhang

**Affiliations:** ^1^ Department of Urology, The First Affiliated Hospital of Soochow University, Suzhou, China; ^2^ Department of Reproductive Medicine Center, The First Affiliated Hospital of Soochow University, Suzhou, China

**Keywords:** muscle-invasive bladder cancer, disitamab vedotin, gemcitabine, cisplatin, immunotherapy, pathological complete response rate

## Abstract

**Introduction:**

Muscle-invasive bladder cancer (MIBC) is an aggressive bladder cancer characterized by invasion of the muscular bladder wall, often necessitating a multimodal treatment approach for optimal outcomes. This study aimed to compare the real-world efficacy and safety of disitamab vedotin (RC48), an antibody-drug conjugate (ADC), combined immunotherapy targeting programmed cell death protein-1 (PD-1), against the gemcitabine and cisplatin (GC) regimen with PD-1 immunotherapy in the treatment of MIBC.

**Methods:**

This single-center, retrospective study was conducted at the First Affiliated Hospital of Soochow University and included 38 patients with MIBC treated with either RC48 plus immunotherapy or GC regimen plus immunotherapy, between January 2022 and December 2023. Patients were divided into two groups: the RC48 with immunotherapy (ADC + PD-1) group and the GC regimen with immunotherapy (GC + PD-1) group. Efficacy was evaluated based on their pathological complete response rates (PCRR) and pathological downstaging rates (PDR). Adverse events (AEs) were assessed to compare safety profiles.

**Results:**

Of the 38 patients, 17 were in the ADC + PD-1 group and 21 were in the GC + PD-1 group. The PCRR was significantly higher in the ADC + PD-1 group (82.35%, 14/17) compared to the GC + PD-1 group (47.62%, 10/21; *P* = 0.043). The PDR was also higher in the ADC + PD-1 group (94.12%, 16/17) than in the GC + PD-1 group (80.95%, 17/21), although the difference was not statistically significant (*P* = 0.355). No serious allergic reactions or fatal AEs were reported in either group. No Grade 4 AEs were reported, while Grade 3 AEs occurred at a rate of 5.71% in the ADC + PD-1 group and 12.20% in the GC + PD-1 group (*P* = 0.260).

**Conclusion:**

RC48 combined with immunotherapy demonstrated a significantly higher PCRR compared to the GC regimen with immunotherapy, while maintaining a comparable safety profile. These findings highlight the potential of RC48 combined with immunotherapy as an effective treatment option for MIBC in clinical practice.

## 1 Introduction

Bladder cancer is among the most common malignant diseases of the urinary system that significantly affects the lifespan and quality of life (1). Muscle-invasive bladder cancer (MIBC) poses substantial difficulties due to its aggressive nature and propensity for metastasis (2). At present, the standard treatment for MIBC (T2-T4aN0M0) involves administering at least three cycles of platinum-based neoadjuvant chemotherapy (NAC) followed by radical cystectomy (RC) (3, 4). Despite this intensive treatment strategy (NAC + RC), over 40% of patients with MIBC face recurrence or mortality within 3 years (3, 4). This underscores the critical need for innovative pre-surgery therapeutic strategies to enhance outcomes for patients.

Immune checkpoint inhibitors (ICIs), including agents targeting programmed cell death protein-1 (PD-1) and programmed death ligand-1 (PD-L1), have recently demonstrated promising survival benefits in patients with locally advanced and metastatic bladder cancer (5, 6). Our previous study revealed that combining gemcitabine and cisplatin (GC) regimens with immunotherapy significantly improved the pathological complete response rates (PCRR) and pathological downstaging rates (PDR) while maintaining a favorable safety profile (1). The overexpression of human epidermal growth factor receptor 2 (HER2) is closely associated with bladder tumor development and progression (7). Consequently, HER2-targeted therapies, particularly antibody-drug conjugates (ADCs) such as disitamab vedotin (RC48), have garnered attention for their demonstrated efficacy and safety in treating locally advanced or metastatic bladder cancer (8–10). Recent studies suggest that combining RC48 with immunotherapy may enhance treatment outcomes (11, 12). And we hypothesized that the combination of RC48 with PD-1 inhibitors would be superior to the traditional GC regimen due to the specific mechanisms of action involved. RC48, as an antibody-drug conjugate, specifically targets HER2-expressing cancer cells, providing a more directed therapeutic approach and minimizing off-target effects, which could potentially lead to higher efficacy and improved safety profiles (9). Additionally, the combination with PD-1 inhibitors may further enhance anti-tumor activity by promoting a stronger immune-mediated response (12). However, further investigations are needed to compare the effectiveness and safety of ADCs combined with immunotherapy versus GC regimen with immunotherapy in management of MIBC.

This study aimed to evaluate the real-world efficacy and safety of RC48 combined with immunotherapy compared to a GC regimen combined with immunotherapy for treatment of MIBC. We retrospectively reviewed the clinical data of patients with MIBC who were treated for MIBC, and who received either ADC combined with immunotherapy or a GC regimen combined with immunotherapy. This study explored the treatment efficacy and adverse events (AEs), providing additional evidence and clinical guidance for the application of RC48 with immunotherapy in the treatment of MIBC.

## 2 Materials and methods

### 2.1 Study design and patient selection

This study retrospectively enrolled sequential individuals diagnosed with MIBC from the Department of Urology at the First Affiliated Hospital of Soochow University. These patients underwent treatment with either an ADC or a GC regimen combined with immunotherapy between January 2022 and December 2023. Eligibility criteria included: 1) Pathologically confirmed MIBC with immunohistochemically demonstrated positivity for HER2 and PD-1 following diagnostic resection of bladder tumor; 2) No prior systemic therapy; 3) Measurable lesions per Response Evaluation Criteria in Solid Tumors (RECIST) v1.1 criteria; 4) Imaging such as ultrasound, computed tomography (CT), multiparametric magnetic resonance imaging (MRI), or positron emission tomography (PET)-MRI showing no distant metastases, and clinical staging of T2-T4aN0M0; 5) Completion of at least three cycles of neoadjuvant therapy; 6) No other concurrent malignancies and no severe impairment of heart, liver, or kidney functions. The exclusion criteria included: 1) Previous treatment with other targeted or immune therapies; 2) Presence of additional malignancies; 3) Serious systemic diseases; 4) Incomplete clinical or pathological data. The patients were categorized into either the RC48 plus immunotherapy group (ADC + PD-1) or the GC regimen plus immunotherapy group (GC + PD-1). The follow-up data collected included basic patient information such as age, sex, Eastern Cooperative Oncology Group (ECOG) performance status, clinical TNM stage, and histological grade. This research complied with the 2013 updated Declaration of Helsinki and received ethical approval from the Institutional Review Board of the First Affiliated Hospital of Soochow University (Approval ID: No.504, 2024). Written informed consent was secured from all the participants prior to inclusion.

### 2.2 Treatment regimen

As mentioned in Section 2.1, the included patients (n=38) were divided into two groups based on the treatment regimen: 17 patients were assigned to the ADC + PD-1 group, with eight receiving tislelizumab and nine receiving toripalimab; and 21 patients were assigned to GC + PD-1 group, with nine receiving tislelizumab and 12 receiving toripalimab.

In the ADC + PD-1 group, disitamab vedotin was administered at 120 mg via intravenous infusion on day 1, followed by immunotherapy with either toripalimab (200 mg) or tislelizumab (240 mg) intravenously on day 2. In the GC + PD-1 group, the regimen included gemcitabine at 1000 mg/m² intravenously on days 1 and 8 and cisplatin at 70 mg/m² intravenously on days 2 and 3 to minimize chemotherapy-related reactions and enhance tolerance. Immunotherapy for this group involved toripalimab at 200 mg or tislelizumab at 240 mg administered intravenously on day 8. Each cycle lasted 21 days, with patients undergoing at least three cycles before RC. Routine blood tests, biochemical tests, thyroid function assessments, and levels of cardiac markers and adrenal hormones were monitored before each treatment. Additionally, multiparametric MRI or PET/MRI was performed prior to surgery, with clinical data collected during outpatient visits, from hospital records, or through telephonic follow-ups.

### 2.3 Observation metrics

The primary endpoints of this study were the postoperative PDR and PCRR. Pathological downstaging was defined as the absence of bladder muscle invasion and lymph node metastasis (≤ ypT1N0M0) in the postoperative pathology, while a pathological complete response was indicated by no residual tumor (ypT0N0M0) in the postoperative pathology. The secondary endpoints involved evaluating changes in the target lesions via multiparametric MRI or PET/MRI after completing the scheduled cycles of the RC48 or GC regimen combined with immunotherapy. Efficacy was evaluated based on RECIST v1.1 criteria, including complete response (CR), partial response (PR), stable disease (SD), and disease progression (DP). Objective response (OR) was defined as CR plus PR, and disease control included CR, PR, and SD. Additionally, AEs were evaluated to assess safety. AEs were recorded and assessed according to the Common Terminology Criteria for Adverse Events (CTCAE) version 5.0 during neoadjuvant therapy (13).

### 2.4 Statistical analyses

Statistical analyses were conducted using R version 4.2.1, employing the ‘stats [4.2.1]’ library. Categorical variables were summarized as proportions (%), whereas continuous variables were reported as mean ± standard deviation. For quantitative data, a t-test was utilized when the conditions of normality and equal variance were met. Welch’s t-test was applied for normally distributed data that did not satisfy the assumption of equal variance. The Wilcoxon test was used for data that did not follow a normal distribution. For categorical data, Fisher’s exact test was employed if the expected frequency was below 1 or the overall sample size was fewer than 40 participants. A P-value of less than 0.05 was considered statistically significant.

## 3 Results

### 3.1 Clinicopathological statistics

As of December 31, 2023, a total of 38 patients were enrolled in the study: 17 in the ADC + PD-1 group and 21 in the GC + PD-1 group. There were no statistically significant differences between the groups in terms of sex, age, body mass index (BMI), hypertension (HBP), diabetes mellitus (DM), smoking history, ECOG performance status, histological grading, HER2 expression, or clinical T (cT) stage. The baseline characteristics of the two groups are summarized in **Table 1**. According to preoperative clinical T staging, 58.82% (10/17) of the patients in the ADC + PD-1 group and 61.90% (13/21) of the patients in the GC + PD-1 group had tumors staged at cT3 or higher.

**Table 1:** Comparison of clinicopathological characteristics between the ADC+PD-1 and GC+PD-1 groups.

Characteristics	GC+PD1 Group	ADC+PD1 Group	*P*-value
n	21	17	
Sex, n (%)			0.491
Men n (%)	16 (76.19)	11 (64.71)	
Women n (%)	5 (23.81)	6 (35.29)	
Age (year), mean ± sd	67.95 ± 7.90	72.94 ± 8.16	0.064
BMI (kg/m^2^), mean ± sd	23.28 ± 3.09	21.70 ± 2.61	0.100
HBP, n (%)			0.185
Yes	6 (28.57)	9 (52.94)	
No	15 (71.43)	8 (47.06)	
DM, n (%)			0.426
Yes	3 (14.29)	5 (29.41)	
No	18 (85.71)	12 (70.59)	
Smoking History, n (%)			0.509
Yes	7 (33.33)	8 (47.06)	
No	14 (66.67)	9 (52.94)	
ECOG Score, n (%)			0.852
0	9 (42.86)	8 (47.06)	
1	8 (38.10)	5 (29.41)	
2	2 (9.52)	3 (17.65)	
3	2 (9.52)	1 (5.88)	
Histological grade, n (%)			1.000
High Grade	19 (90.48)	16 (94.12)	
Low Grade	2 (9.52)	1 (5.88)	
HER2 expression, n (%)			0.255
1+	1 (4.76)	1 (5.88)	
2+	16 (76.19)	9 (52.94)	
3+	4 (19.05)	7 (41.18)	
cT staging, n (%)			0.844
cT2	8 (38.10)	7 (41.18)	
cT3	6 (28.57)	6 (35.29)	
cT4a	7 (33.33)	4 (23.53)	
Combined immunotherapy drugs, n (%)			1.000
Toripalimab	12 (57.14)	9 (52.94)	
Tisleizumab	9 (42.86)	8 (47.06)	

BMI, body mass index; HBP, high blood pressure; DM, diabetes mellitus; ECOG, Eastern Cooperative Oncology Group; HER2, human epidermal growth factor receptor 2; GC, gemcitabine and cisplatin; PD1, programmed death protein 1; ADC, antibody-drug conjugate; sd, standard deviation.

### 3.2 Comparison of PDR and PCRR between the two groups

In the ADC + PD-1 group, 14 patients achieved complete remission (ypT0N0M0) and two patients achieved partial remission (ypT1N0M0), resulting in a PDR of 94.12% and a PCRR of 82.35%. In contrast, in the GC + PD-1 group, 10 patients achieved complete remission (ypT0N0M0) and seven achieved partial remission (ypT1N0M0), leading to a PDR of 80.95% and a PCRR of 47.62%. Additionally, in the ADC + PD-1 group, 14 patients achieved CR, 2 achieved PR, 1 had SD, and none experienced DP, resulting in an OR rate (ORR) of 94.12% and a disease control rate (DCR) of 100%. In the GC + PD-1 group, 10 patients achieved CR, 6 achieved PR, and 5 had SD, with no DP, leading to an ORR of 76.19% and a DCR of 100%. The PCRR and CR in the ADC + PD-1 group were significantly higher than those in the GC + PD-1 group (*P* < 0.05). However, there were no significant differences in the PDR, PR, SD, or ORR between the two groups (*P* > 0.05) (**Table 2**).

**Table 2:** Comparison of PDR and PCRR between the ADC+PD-1 and GC+PD-1 groups.

Characteristics	GC+PD1 Group	ADC+PD1 Group	*P*-value
n	21	17	
Pathological outcome, n (%)			0.117
ypT0	10 (47.62)	14 (82.35)	
ypT1	7 (33.33)	2 (11.76)	
≥ypT2	4 (19.05)	1 (5.88)	
PCRR, n (%)			0.043
Yes	10 (47.62)	14 (82.35)	
No	11 (52.38)	3 (17.65)	
PDR, n (%)			0.355
Yes	17 (80.95)	16 (94.12)	
No	4 (19.05)	1 (5.88)	
CR, n (%)			0.043
Yes	10 (47.62)	14 (82.35)	
No	11 (52.38)	3 (17.65)	
PR, n (%)			0.257
Yes	6 (28.57)	2 (11.76)	
No	15 (71.43)	15 (88.24)	
SD, n (%)			0.197
Yes	5 (23.8)	1 (5.9)	
No	16 (76.2)	16 (94.1)	
Objective Response, n (%)			0.197
Yes	16 (76.19)	16 (94.12)	
No	5 (23.81)	1 (5.88)	

PCRR, pathological complete response rate; PDR, pathological downstaging rate; GC, gemcitabine and cisplatin; PD1, programmed death protein 1; ADC, antibody drug conjugate; CR, complete response; PR, partial response; SD, stable disease.

### 3.3 Summary of treatment-related adverse events in MIBC patients

No severe allergic reactions or fatal AEs were reported in either group and there were no grade 4 AEs. The incidence of AEs was comparable between the ADC + PD-1 and GC + PD-1 groups. The incidence of grade 3 AEs was 5.71% (5/24) in the ADC + PD-1 group and 12.20% (10/82) in the GC + PD-1 group; however, the difference was not statistically significant (*P* > 0.05; **Table 3**).

**Table 3:** Comparison of treatment-related adverse events between the ADC+PD-1 and GC+PD-1 Groups.

Characteristics	GC+PD1	ADC+PD1	*P*-value
n	21	17	
Peripheral sensory neuropathy, n (%)			0.803
0	17 (81)	15 (88.2)	
1	3 (14.3)	1 (5.9)	
2	1 (4.8)	1 (5.9)	
Alopecia, n (%)			0.678
0	18 (85.7)	13 (76.5)	
1	3 (14.3)	4 (23.5)	
Asthenia, n (%)			0.708
0	17 (81)	11 (64.7)	
1	1 (4.8)	3 (17.6)	
2	1 (4.8)	1 (5.9)	
3	2 (9.5)	2 (11.8)	
Decreased appetite, n (%)			0.710
0	13 (61.9)	9 (52.9)	
1	4 (19)	6 (35.3)	
2	3 (14.3)	2 (11.8)	
3	1 (4.8)	0 (0)	
Weight decrease, n (%)			0.299
0	18 (85.7)	11 (64.7)	
1	2 (9.5)	5 (29.4)	
2	1 (4.8)	1 (5.9)	
Anemia, n (%)			0.759
0	15 (71.4)	15 (88.2)	
1	1 (4.8)	0 (0)	
2	2 (9.5)	1 (5.9)	
3	3 (14.3)	1 (5.9)	
Nausea, n (%)			0.514
0	13 (61.9)	13 (76.5)	
1	3 (14.3)	2 (11.8)	
2	2 (9.5)	2 (11.8)	
3	3 (14.3)	0 (0)	
Gamma glutamyl transpeptidase, n (%)			0.580
0	17 (81)	14 (82.4)	
1	3 (14.3)	1 (5.9)	
2	1 (4.8)	2 (11.8)	
Aspartate aminotransferase level increased, n (%)			0.355
0	16 (76.2)	16 (94.1)	
1	4 (19)	1 (5.9)	
2	1 (4.8)	0 (0)	
Pruritus, n (%)			0.514
0	16 (76.2)	10 (58.8)	
1	3 (14.3)	5 (29.4)	
2	1 (4.8)	2 (11.8)	
3	1 (4.8)	0 (0)	
Leukopenia, n (%)			0.776
0	16 (76.2)	11 (64.7)	
1	3 (14.3)	4 (23.5)	
2	2 (9.5)	2 (11.8)	
Alanine aminotransferase level increased, n (%)			0.119
0	14 (66.7)	15 (88.2)	
1	6 (28.6)	1 (5.9)	
2	1 (4.8)	1 (5.9)	
Blood triglycerides increased, n (%)			0.613
0	18 (85.7)	16 (94.1)	
1	3 (14.3)	1 (5.9)	
Neutrophil count decreased, n (%)			0.249
0	17 (81)	11 (64.7)	
1	3 (14.3)	6 (35.3)	
2	1 (4.8)	0 (0)	
Blood glucose increased, n (%)			0.321
0	19 (90.5)	12 (70.6)	
1	1 (4.8)	3 (17.6)	
2	1 (4.8)	2 (11.8)	
Platelet count decreased, n (%)			0.072
0	17 (81)	15 (88.2)	
1	4 (19)	0 (0)	
2	0 (0)	1 (5.9)	
3	0 (0)	1 (5.9)	
Blood bilirubin increased, n (%)			0.705
Total adverse events, n (%)			0.260
Grade 1–2	72 (87.80)	66 (94.29)	
Grade 3	10 (12.20)	4 (5.71)	

GC, gemcitabine and cisplatin; PD1, programmed death protein 1; ADC, antibody drug conjugates.

## 4 Discussion

Currently, platinum-based NAC is recognized as the preferred approach for surgically resectable MIBC. This approach is an A-level recommendation in several authoritative urological guidelines, including those of the European Association of Urology, American Urological Association, and Canadian Urological Association, guiding clinical practice (14, 15). Frequently applied NAC regimens include MVAC (methotrexate, vinblastine, adriamycin and cisplatin) and GC (3, 4, 16, 17). However, owing to high toxicity and limited tolerability of the MVAC regimen, it has been largely replaced by the GC regimen (18, 19). The SWOG-8710 trial highlighted a 5-year survival rate of 57% in the NAC-treated cohort compared to 38% in patients undergoing surgery alone, with PCRR of 38% and 15%, respectively (20). A meta-analysis conducted in the United Kingdom showed that platinum-based NAC significantly improved overall survival (OS) in patients with MIBC, reducing the risk of death by 13% and increasing the 5-year survival rate by 5% (21). Despite these advantages, the proportion of patients receiving NAC remains low, primarily due to factors such as advanced age, impaired renal function, poor physical condition, and other comorbidities that render them unable to tolerate platinum-based chemotherapy regimens. A study by Rose et al. (22) on the efficacy of immunotherapy (pembrolizumab) combined with chemotherapy (GC regimen) as neoadjuvant treatment for MIBC reported a PCRR of 36% and a PDR of 56%. The NEODURVARIB study analyzed the efficacy of neoadjuvant immunotherapy (durvalumab) combined with targeted therapy (olaparib) in patients with bladder cancer, including 29 patients with MIBC, and found a PCRR of 50% (23). Our previous study involving 53 patients with MIBC also demonstrated that NAC combined with immunotherapy led to higher PCRR (51.85% vs. 23.08%, *P*=0.031) and PDR (77.78% vs. 42.31%, *P*=0.008) than NAC alone (1). The advent of new targeted therapies has opened additional treatment options for patients with bladder cancer.

ADCs are a novel targeted therapy comprising a humanized monoclonal antibody with high affinity and specificity, a highly stable linker, and a potent cytotoxic payload. They exert antitumor effects while minimizing damage to normal tissues (24, 25). ADC monotherapy or ADC combined with immunotherapy plays a crucial role in the treatment of locally advanced or metastatic urothelial carcinoma. In the RC48-C005 study, 43 patients with HER2-overexpressing metastatic urothelial carcinoma received disitamab vedotin, achieving an ORR of nearly 50%, median progression free survival (PFS) of 6.9 months, and median OS of 13.9 months (9). The RC48-C009 study included 64 patients with HER2-overexpressing metastatic urothelial carcinoma who had previously failed to respond to multiple lines of chemotherapy. In this study, 85.9% of the patients had previously received second-line or more systemic treatments. Those treated with RC48 had a median duration of response (DOR) of 8.3 months, a DCR of 76.6%, evident benefits across subgroups, and a low rate of severe AEs (26). The RC48-C014 study demonstrated that RC48 combined with toripalimab had promising efficacy in locally advanced or metastatic bladder cancer, regardless of HER2 expression status, with an ORR of 75%, a complete response rate of 15%, and a DCR of 95%. In a retrospective study by Wei et al. (27), nine patients with stage T2-4aN0-3M0 disease from two centers were included, with five receiving RC48 combined with tislelizumab and four receiving toripalimab. The study reported an overall ORR of 88.9%, with five patients achieving CR, three achieving PR, one experiencing DP, a median progression-free survival (PFS) of 12.0 months, and a low incidence of ≥grade 3 events. Xu et al. (12) conducted a retrospective study involving 38 patients with locally advanced or metastatic urothelial carcinoma from multiple centers. Of these, eight received RC48 monotherapy, and 30 were treated with RC48 combined with tislelizumab or toripalimab. The overall ORR was 63.2%, with a DCR of 89.5%, a median PFS of 8.2 months, an unreached median OS, and a 12-month OS rate of 76.7%. In patients treated with RC48 plus immunotherapy, the ORR, DCR, and PFS were 66.7%, 90.0%, and 8.2 months, respectively. Additionally, ADCs exhibit a “bystander effect” which can alter the tumor microenvironment, enhancing the efficacy of other anti-tumor drugs (28). This aligns with the findings of this study, where only patients with HER2 1+ expression in the ADC + PD-1 group achieved a PCR following treatment. However, due to the small sample size, further studies are required to confirm these findings. There is a relative paucity of studies on neoadjuvant RC48 combined with immunotherapy, particularly large-scale studies, both domestically and internationally. Based on the promising results of RC48 as monotherapy and in combination with immunotherapy in advanced or metastatic bladder cancer, along with preliminary exploration in neoadjuvant settings, our center is among the first to report the efficacy and safety of RC48 combined with immunotherapy compared to the GC regimen plus immunotherapy for MIBC. The primary endpoints were the PCRR and PDR. Results showed a PCRR of 82.35% and a PDR of 94.12% in the ADC + PD-1 group, compared to a PCRR of 47.62% and a PDR of 80.95% in the GC + PD-1 group. The difference in PCRR between the groups was statistically significant, and although the difference in PDR was not statistically significant, the ADC combined with the PD-1 inhibitor neoadjuvant strategy showed a higher PDR than the GC NAC regimen. These findings are consistent with similar clinical studies (15, 24), providing valuable guidance for clinical practice.

The safety analysis indicated no significant increase in the incidence of AEs in the ADC + PD-1 group compared with to the GC+PD-1 group. Thus, it appears that ADC combined with immunotherapy does not increase the incidence of AEs compared to GC combined with immunotherapy. However, due to the lack of cohorts studying only neoadjuvant targeted therapy or immunotherapy, further research is required to confirm these conclusions.

This study provides a preliminary analysis of the efficacy and safety of ADC combined with a PD-1 inhibitor in the neoadjuvant treatment of bladder cancer, using GC combined with immunotherapy as a control. This comparative approach yields more robust and persuasive results than single-cohort studies. These findings indicate a high rate of PCR and downstaging, providing a theoretical basis for clinical practice. Additionally, while the study is ongoing, the preliminary results suggest that ADC combined with a PD-1 inhibitor is an effective neoadjuvant treatment for bladder cancer, with manageable safety profiles. Additional cases will be included in future analyses for further validate these findings.

However, this study has some limitations. First, this was a single-center retrospective study with a relatively small sample size, which unavoidably introduced a selection bias. Second, the center had few patients with bladder cancer receiving disitamab vedotin monotherapy or immunotherapy alone as neoadjuvant treatment; therefore, relevant cohorts were not included for comparison. Third, the retrospective nature of the study may lead to incomplete data collection and potential inaccuracies in patient records. Finally, the follow-up period in this study was short, preventing the assessment of long-term survival outcomes. To enhance the scientific value and applicability of this research, future studies should focus on prospective, larger-scale trials.

In summary, the first domestically approved ADC (disitamab vedotin) combined with PD-1 inhibitors (tislelizumab and toripalimab) demonstrates a higher pathological complete response rate and pathological downstaging rate in the neoadjuvant treatment of bladder cancer than traditional GC chemotherapy. In addition, the associated adverse reactions are manageable.

## Data Availability

The datasets presented in this study can be found in online repositories. The names of the repository/repositories and accession number(s) can be found below: https://figshare.com/s/c09971ae32a9be5b4305.
